# Speech-specific audiovisual integration modulates induced theta-band oscillations

**DOI:** 10.1371/journal.pone.0219744

**Published:** 2019-07-16

**Authors:** Alma Lindborg, Martijn Baart, Jeroen J. Stekelenburg, Jean Vroomen, Tobias S. Andersen

**Affiliations:** 1 Section for Cognitive Systems, DTU Compute, Technical University of Denmark, Lyngby, Denmark; 2 Department of Cognitive Neuropsychology, Tilburg University, Tilburg, The Netherlands; 3 BCBL. Basque Center on Cognition, Brain and Language, Donostia, Spain; Harvard Medical School, UNITED STATES

## Abstract

Speech perception is influenced by vision through a process of audiovisual integration. This is demonstrated by the McGurk illusion where visual speech (for example /ga/) dubbed with incongruent auditory speech (such as /ba/) leads to a modified auditory percept (/da/). Recent studies have indicated that perception of the incongruent speech stimuli used in McGurk paradigms involves mechanisms of both general and audiovisual speech specific mismatch processing and that general mismatch processing modulates induced theta-band (4–8 Hz) oscillations. Here, we investigated whether the theta modulation merely reflects mismatch processing or, alternatively, audiovisual integration of speech. We used electroencephalographic recordings from two previously published studies using audiovisual sine-wave speech (SWS), a spectrally degraded speech signal sounding nonsensical to naïve perceivers but perceived as speech by informed subjects. Earlier studies have shown that informed, but not naïve subjects integrate SWS phonetically with visual speech. In an N1/P2 event-related potential paradigm, we found a significant difference in theta-band activity between informed and naïve perceivers of audiovisual speech, suggesting that audiovisual integration modulates induced theta-band oscillations. In a McGurk mismatch negativity paradigm (MMN) where infrequent McGurk stimuli were embedded in a sequence of frequent audio-visually congruent stimuli we found no difference between congruent and McGurk stimuli. The infrequent stimuli in this paradigm are violating both the general prediction of stimulus content, and that of audiovisual congruence. Hence, we found no support for the hypothesis that audiovisual mismatch modulates induced theta-band oscillations. We also did not find any effects of audiovisual integration in the MMN paradigm, possibly due to the experimental design.

## Introduction

Speech is perceived with both audition and vision. Seeing the face of the speaker improves comprehension, particularly if the auditory signal is weak or degraded [1], and speeds up the neural processing of speech [2].

The McGurk effect–where dubbing an auditory syllable onto an incongruent speech video leads to a modified auditory percept (eg. auditory /ba/ and visual /ga/ leading to the perception of /da/)–is a striking behavioural demonstration of audiovisual (AV) integration in speech perception [3]. Ever since its discovery, the McGurk effect has widely been used as a measure of audiovisual integration [4–8]. However, more recently it has been argued that the perceptual fusion of incongruent audiovisual stimuli is different from that of congruent, naturally occurring speech, as it requires incongruence processing in addition to the mechanism of audiovisual integration [9,10].

Electroencephalography (EEG) studies have shown that visual speech modulates the neural processing of speech as reflected in event-related potentials (ERPs), both by shortening the latency and decreasing the amplitude of the N1 and P2 peaks, occurring within approximately 250 ms. after stimulus onset [11–16]. Moreover, both auditory [17–19] and audiovisual [20,21] speech has been shown to modulate cortical oscillations, with the consistent finding that low-frequency oscillations (2–8 Hz) entrain to the speech envelope, possibly serving as a basis for temporal organization of the neural processing of speech [22,23]. However, it has been contended that other features in the speech signal (e.g. spectral and phonetic information) may be more predictive of the brain signal than the envelope [24], and that the frequency of perceptually relevant entrained oscillations may operate on stimulus-specific time scales determined by, e.g., phrasal rate [25].

Cortical oscillations have also received attention within the study of multisensory integration, where it has been suggested that modulations of ongoing oscillatory activity may form a substrate of the communication between distant neural populations which support multisensory integration [26–29]. Such modulations may manifest themselves either in the phase domain (as in the rhythmic entrainment case), or in the amplitude of either *evoked* (phase-locked) or *induced* (non-phase-locked) oscillations.

The first published study of amplitude modulations of induced oscillations in relation to audiovisual integration found that the McGurk illusion is accompanied by a decrease of induced theta-band (4–8 Hz) oscillations [30]. This decrease was centred on frontal and fronto-central sensors and appeared from 200–600 ms. after sound onset. In the study, Keil and colleagues argued that differences in incongruence processing–leading to a resolution of the incongruence in fusion trials but not in the non-fusion trials–were driving the effect, suggesting it was a case of general mismatch processing which is also, for example, indexed by the auditory mismatch negativity (MMN).

This claim has gained further support in two recent studies. First, an fMRI study made a similar claim that the McGurk illusion recruits general-purpose conflict areas as well as specialized audiovisual speech conflict areas in the brain [31]. Secondly, an EEG study specifically targeted at the theta band found a difference in total power between AV congruent and McGurk trials which had a topographical correlation to the theta-band effect of incongruence in a Stroop task, suggesting activation of a general "conflict processing network" in both tasks [32].

It is worthy to note that the paradigms which in the literature are suggested to produce the same theta-band effect are quite different from one another. Whereas the McGurk effect would produce a mismatch signal because the audio and visual components are incongruent, the MMN is a an auditory memory related mismatch signal and the Stroop paradigm indexes a non-speech semantic incongruence. Thus, if these effects are comparable, it means that the theta-band modulation found by Keil et al. (2012) is produced by a general-purpose conflict processing network.

However, an alternative explanation of the theta-band suppression could be that, rather than reflecting either general or specific mismatch processing, it stems from the audiovisual integration that is present in the fusion trials but absent in non-fusion trials. Although both mismatch processing and audiovisual integration could modulate the theta band at the same time and the hypotheses are thus not mutually exclusive, we argue that they should both be considered.

Here we will contrast the two explanations by testing the following competing hypotheses: The suppression of theta-band oscillations is driven by audiovisual (phonetic) *integration* of speech; or alternatively it is related to *mismatch* processing in either the AV incongruence or task-general sense. A useful stimulus for this purpose is sine-wave speech (SWS), a form of spectrally degraded speech in which the speech signal is replaced by sinusoids at the centre frequencies of the first three formants [33]. To a naïve listener, SWS sounds like computer beeps or whistles, but subjects who are informed that SWS is derived from speech can perceive its phonetic content. When subjects are naïve to the speech origin of the sound, they are in *non-speech mode* (NSM), but once they perceive SWS as speech, they cannot revert to the naïve state and are in *speech mode* (SM). When SWS is combined with visual speech, it has been found that SM perceivers get a McGurk illusion whereas NSM perceivers do not [34]. Moreover, EEG studies have shown that audiovisual SWS can suppress the P2 component and induce a McGurk-MMN in a similar manner as natural AV speech for SM, but not NSM perceivers [13,35]. On the other hand, SM and NSM perceivers get a similar multisensory detection advantage in AV detection tasks [36] and they show similar visually-driven modulation of the N1 EEG component [13]. This indicates that early, low-level processing as indexed by the N1 and AV detection benefit is unaffected by perceptual mode, whereas phonetic processing as indexed by integration of phonetic information, McGurk-MMN and P2 is modulated by perceptual mode.

Using the framework of sine-wave speech, we can overcome the confounding of integration and mismatch effects inherent in natural speech McGurk paradigms. In order to consider the *integration hypothesis* and the *mismatch hypothesis* separately, we will use EEG recordings from two previously published studies.

1. The N1/P2 dataset contains EEG from a SWS experiment designed to investigate the influence of speech-specific audiovisual integration on the N1 and P2 components of ERPs [13]. This study recorded EEG data from subjects in a passive stimulation paradigm. Subjects perceived congruent and incongruent audiovisual, as well as unimodal auditory and visual SWS stimuli in SM and NSM. The audiovisual stimuli were identical for the first syllable (~270 ms), after which the incongruent stimuli differed from congruent.
2. The MMN dataset comprises EEG recordings from a McGurk mismatch negativity (McGurk-MMN) study [35]. When McGurk stimuli are presented within a sequence of acoustically identical but audio-visually congruent stimuli, they will elicit a mismatch signal known as the McGurk-MMN–an enhanced negativity in the evoked potential for deviant compared to standard trials occurring about 150–250 ms after mismatch onset [35,37]. The authors found that McGurk-MMN occurred only for subjects in SM [35].

According to the *integration hypothesis*, audiovisual integration would suppress theta oscillations. Thus, the integration hypothesis predicts that there is a SM < NSM difference for the audiovisual conditions in the N1/P2 dataset, since SM subjects integrated the stimuli whereas NSM subjects did not. Moreover, this difference should not be present in the unimodal conditions. Since the audiovisual conditions in the N1/P2 study contain both trials where stimuli were audio-visually integrated and trials where they were not integrated, and since congruent and incongruent stimuli are identical until the second syllable, it is not clear whether the integration hypothesis would predict a notable difference between congruent and incongruent stimuli for SM perceivers. Moreover, the integration hypothesis would also predict an integration effect in the MMN dataset, again as SM < NSM.

According to the *mismatch hypothesis*, a mismatch signal is elicited when audiovisual incongruence is perceived, resulting in higher theta power compared to congruent trials. The mismatch hypothesis thus predicts a Deviant > Standard difference in the MMN dataset. In line with the reasoning by Morís Fernández et al in [31], this mismatch signal could either stem from a general-purpose mismatch mechanism, or a specialized audiovisual conflict mechanism. Arguably, both of these are captured in the McGurk-MMN paradigm. First, the main component of the McGurk-MMN has a high spatiotemporal resemblance to the auditory MMN [37,38]. This suggests that the McGurk illusion modifies the brain's *auditory* representation of the deviant stimulus, which does not match the expected "standard" sound and hence produces an MMN [37–40]. This makes the McGurk-MMN a good candidate for measuring the general mismatch effect suggested by Keil and colleagues [30]. Secondly, for the modification of the auditory percept to take place, the brain must first resolve the audiovisual incongruence in the deviant stimulus–a process supposedly supported by the specialized audiovisual speech conflict areas [31]. Thus, if the mismatch hypothesis is true, we should replicate the theta effect observed by Keil et al [30] and would thus observe an incongruence or mismatch related difference in theta-band power between standard and deviant trials for SM, but not NSM subjects. Moreover, the mismatch hypothesis predicts no difference between SM and NSM for congruent audiovisual stimuli in the N1/P2 dataset, because they do not contain an audiovisual mismatch. When comparing audiovisual congruent and incongruent trials, it is crucial to note that the incongruence in the audiovisual incongruent trials of the N1/P2 study did not start until the second syllable. Due to the short duration of the SOA the activation evoked/induced by the second syllable lies outside the time range in which it is possible to estimate power in the theta band and hence the mismatch hypothesis does not predict a significant difference between the audiovisual congruent and incongruent conditions in this dataset.

## Methods

### Sine-wave speech N1/P2 dataset

The N1/P2 dataset was originally collected by Baart and colleagues [13]. The experimental procedure is described in detail in the original study but, in short, 28 subjects were randomly assigned to either the Speech Mode (SM) or Non-Speech Mode (NSM) group, with 14 participants in each group. Stimulus material was based on audiovisual recordings of a male speaker pronouncing the Dutch pseudo-words /tabi/ and /tagi/. Vowel to consonant (/a/ to /b/ or /g/) transitions started at 270 and 300 ms, respectively, and the onset of the critical second consonant was 372 ms for /b/ and 428 ms for /g/.

The audio was converted into sine-wave speech and presented in audio-only (Ab and Ag), visual-only (Vb and Vg), audiovisual congruent (AbVb and AgVg) and audiovisual incongruent (AbVg and AgVb) versions. The inter-trial time interval varied randomly between 1 and 2 s.

In order to make sure that participants were paying attention to the visual component of the stimuli, they were engaged in an unrelated visual task consisting of pressing a button whenever an occasional white square appeared on the screen. There was a total of 672 trials: 144 in each condition (A, V, AVC and AVI) and 96 catch trials with the visual task.

The EEG was recorded from 64 electrode locations corresponding to the extended International 10–20 system, at a sampling rate of 512 Hz. The EEG was referenced offline to the average of two additional mastoid electrodes, and two external EOG electrodes were used to register eye-movements.

### Sine-wave speech MMN dataset

The MMN dataset was originally collected by Stekelenburg & Vroomen [35]. In short, 45 subjects were assigned randomly to either an SWS speech mode, SWS non-speech mode, or natural speech group. There were 15 subjects in each group. Stimulus material was based on audiovisual recordings of a male speaker uttering the Dutch pseudowords /omso/ and /onso/, which were delivered with the audio track converted into sine-wave speech for the SWS speech mode and non-speech mode groups, and with the original audio for the natural speech group.

Trials were delivered in an oddball paradigm, in which 1020 of 1200 trials per condition (A, V or AV) were “standards” (An, Vn, and AnVn respectively) and the remaining 180 trials were “deviants” (Am, Vm and AnVm, respectively). Of the 1200 trials, 5% were catch trials, which were excluded from further analysis. Importantly, in the AV condition, there was no difference between standards and deviants in the auditory signal. However, the “deviant” AnVm stimulus is generally heard as /omso/ rather than /onso/ due to the McGurk effect only for listeners in SM (Tuomainen et al., 2005).

The EEG was recorded from 128 locations at a sampling rate of 512 Hz. Electrodes were positioned radially equidistant from the vertex across the scalp according to the BioSemi ABC electrode positioning system. Two mastoid electrodes served as off-line reference and EOG was monitored by bipolar horizontal and vertical electrodes.

### EEG analyses

Analyses of the EEG signal was done in EEGlab [41], FieldTrip [42] and with custom MatLab code.

#### Preprocessing

The EEG signal was first high-pass filtered at 0.5 Hz with a Hamming windowed zero-phase sinc FIR filter of order 1128, following the considerations suggested in [43]. Bad channels were identified by a kurtosis measure (max *z*-score of 5). On average, 3.14 of 64 channels were removed in the N1/P2 dataset (range 0–7 for individual subjects) and 6.60 of 128 channels were removed in the MMN dataset (range 0–13 for individual subjects). The data was subsequently low-pass filtered at 40 Hz with zero-phase FIR filter (Hamming window, order 338) and segmented in epochs of 2 seconds, starting 1 second before and ending 1 second after sound onset. Epochs were baselined to the [-100 ms, 0 ms] interval, and epochs with an absolute amplitude of >150 μV in non-frontal sensors were removed. Subsequently, independent component analysis was run on the baseline corrected data. Artefactual independent components were identified with the help of ICMARC, a semi-automatic classification algorithm using multiple features to classify independent components in multiple artefact classes [44]. Components capturing eye blinks, lateral eye movement, heartbeat and significant muscular activity were identified, resulting in the removal of on average 7.14 independent components for the N1/P2 dataset (ranging from 4 to 14 for individual subjects) and 6.40 independent components for the MMN dataset (ranging from 2 to 18 for individual subjects). After these components were projected out, removed channels were spherically interpolated from the surrounding channels. Before further analysis, the ERP (computed per condition) was subtracted out from each individual trial, removing the evoked component of the signal and leaving only the induced activity.

#### Time-frequency analysis

The EEG signal was transformed into time-frequency space by a wavelet transform, using a family of complex Morlet wavelets of 5 cycles. The transform was computed for the frequency range 2–38 Hz in steps of 2 Hz and time steps of 5 ms.

Bad epochs were rejected based on their maximum log-power value, assuming that trials with an abnormally high (2.5 standard deviations above the individual mean) maximum power at any frequency represent artefacts. Together with the previously applied power threshold (epochs with absolute amplitude of >150 μV in non-frontal sensors), this led to exclusion of 3.32% of the trials for the N1/P2 dataset (ranging from 0 to 10.07% for individual subjects), and 2.77% of the trials for the MMN dataset (ranging between 0.11% and 12.19% for individual subjects).

After removal of bad trials, power values were averaged over trials per subject and condition. A dB baseline was then applied with a 300 ms window (-500 ms to -200 ms, relative to sound onset) serving as baseline.

### Statistical analysis

Statistical comparison of the time-frequency transformed data was done by means of cluster-based permutation tests with Monte Carlo randomization [45]. Between-subjects differences (SM vs NSM) were assessed with independent-samples *t*-statistics and within-subjects differences between conditions were assessed with dependent samples *t*-statistics. Clusters had to contain at least two adjacent sensors and were deemed as significant when the probability of observing a cluster in which shuffling the data led to a larger summed test statistic was below 5%.

For the N1/P2 dataset, between-subjects differences (SM vs. NSM) were tested for each condition (AV Congruent, AV Incongruent, Auditory, Visual), and for the average of both AV conditions. Subsequently, a 2 (Group; Speech mode, Non-speech mode) x 2 (Condition; AV Congruent, AV Incongruent) repeated-measures ANOVA was run on average power in the (time, sensor, frequency) cluster from the combined AV conditions.

For the MMN dataset, Standard vs. Deviant was tested for each group (Speech mode and Non-speech mode), and between-subjects differences were tested for each condition (Standard and Deviant). Only those standard trials which were preceded by a standard trial were considered in the analysis, so that no potential expectation mismatch from a previous deviant trial would carry over to the standard trials.

## Results

### N1/P2 dataset: Lower theta power for speech mode compared to non-speech mode

The integration hypothesis is concerned with audiovisual integration of phonetic features, which occurs in successful McGurk fusions [30] and for congruent audiovisual stimuli but only when SWS is perceived as speech (i.e. for SM subjects [13]). However, phonetic integration does not occur when McGurk trials are not perceptually fused, or when listeners do not perceive SWS as speech (i.e., the NSM subjects). As noted, Keil et al observed that oscillatory power in the theta band was *lower* for successful McGurk fusions than for unsuccessful fusions [30]. Thus, if the integration hypothesis is true, there should be lower induced theta power for SM compared to NSM subjects for congruent and incongruent audiovisual, but not unimodal stimuli.

**Fig 1:**
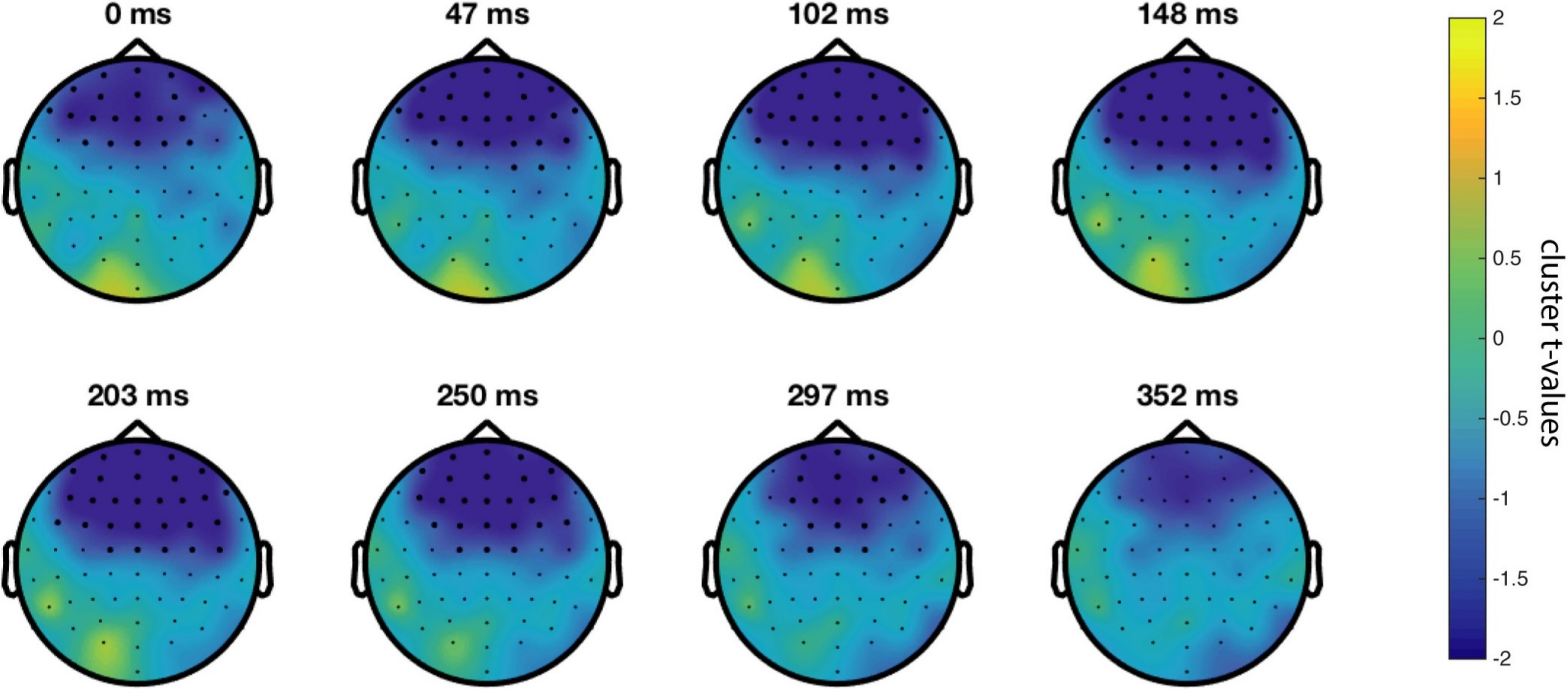
Time evolution of the negative cluster (p = 0.0200) in the 4–8 Hz band for the congruent AV condition. Sensors belonging to the cluster in bold.

**Fig 2:**
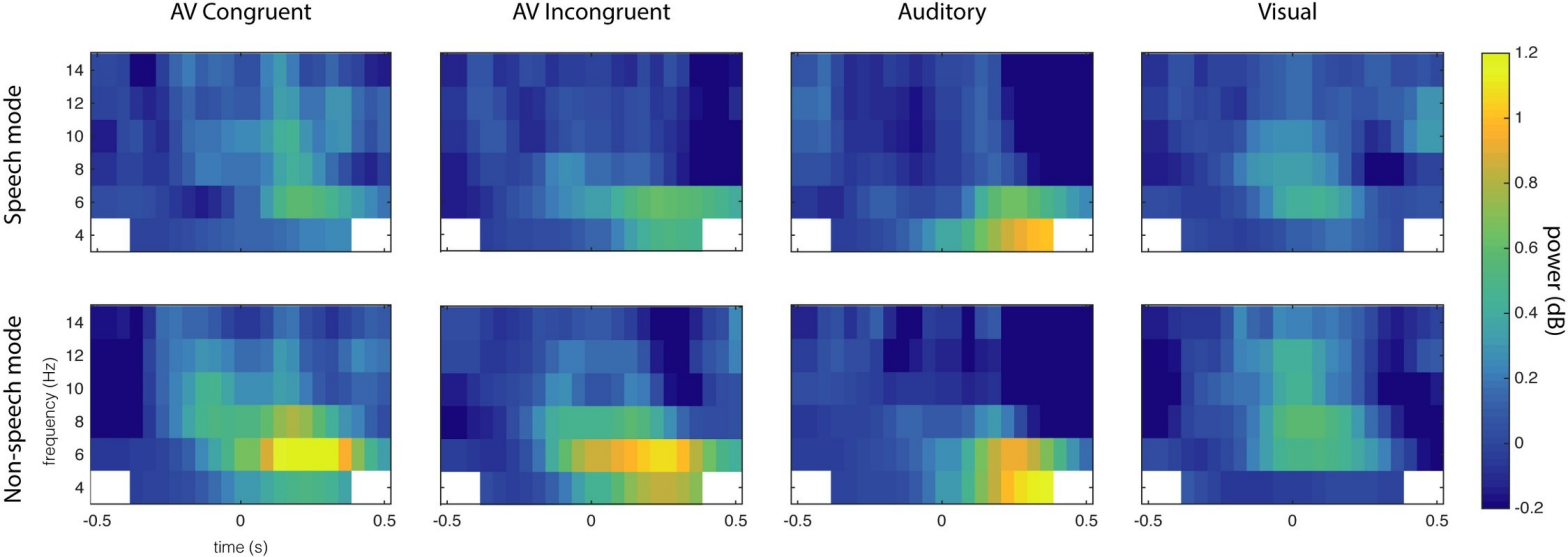
Grand average power by time (x-axis) and frequency (y-axis) for speech mode (upper row) and non-speech mode (lower row) groups in the N1/P2 dataset, at sensor level. In the non-speech mode group, enhanced theta-band activity is observed from around 100 ms to 400 ms. This effect is largely absent in the speech mode group for the audiovisual conditions, with the biggest between-groups difference for Audiovisual Congruent trials.

**Fig 3:**
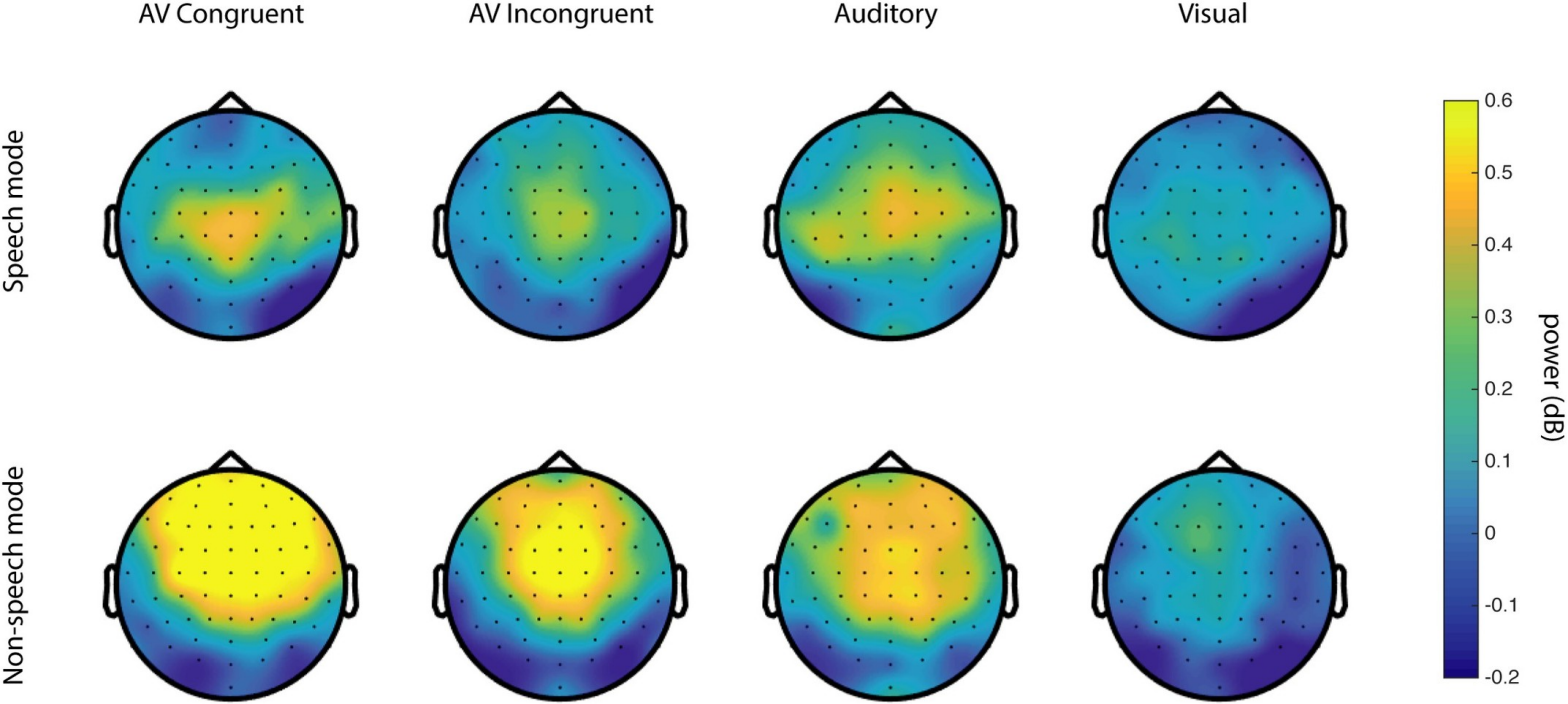
Topographic distribution of grand average 4–8 Hz power for speech mode (top) and non-speech mode (bottom) at 0–300 ms.

We ran a cluster-based permutation test of the SM < NSM hypothesis in the 4–8 Hz frequency range in a 0 to 500 ms window relative to sound onset. Between group permutation tests were run for each condition. For the audiovisual congruent (AVC) stimuli, the SM vs NSM difference was indeed significant (*p* = 0.0200, see Fig 1), but no significant differences were observed for the auditory, visual, and audiovisual incongruent (A, V and AVI) conditions. The significant effect is localized at fronto-central sensors and reaches a maximum number of sensors at around 102–203 ms. The mean induced power for each group and condition is summarized in Figs 2 and 3, showing the time-frequency (Fig 2) and the topographical (Fig 3) dimensions. To make sure that the found effect is not related to an effect in the evoked signal [46], we ran a separate test of the evoked activity only, i.e. the time-frequency transforms of the ERPs, revealing no significant differences between the groups.

**Fig 4:**
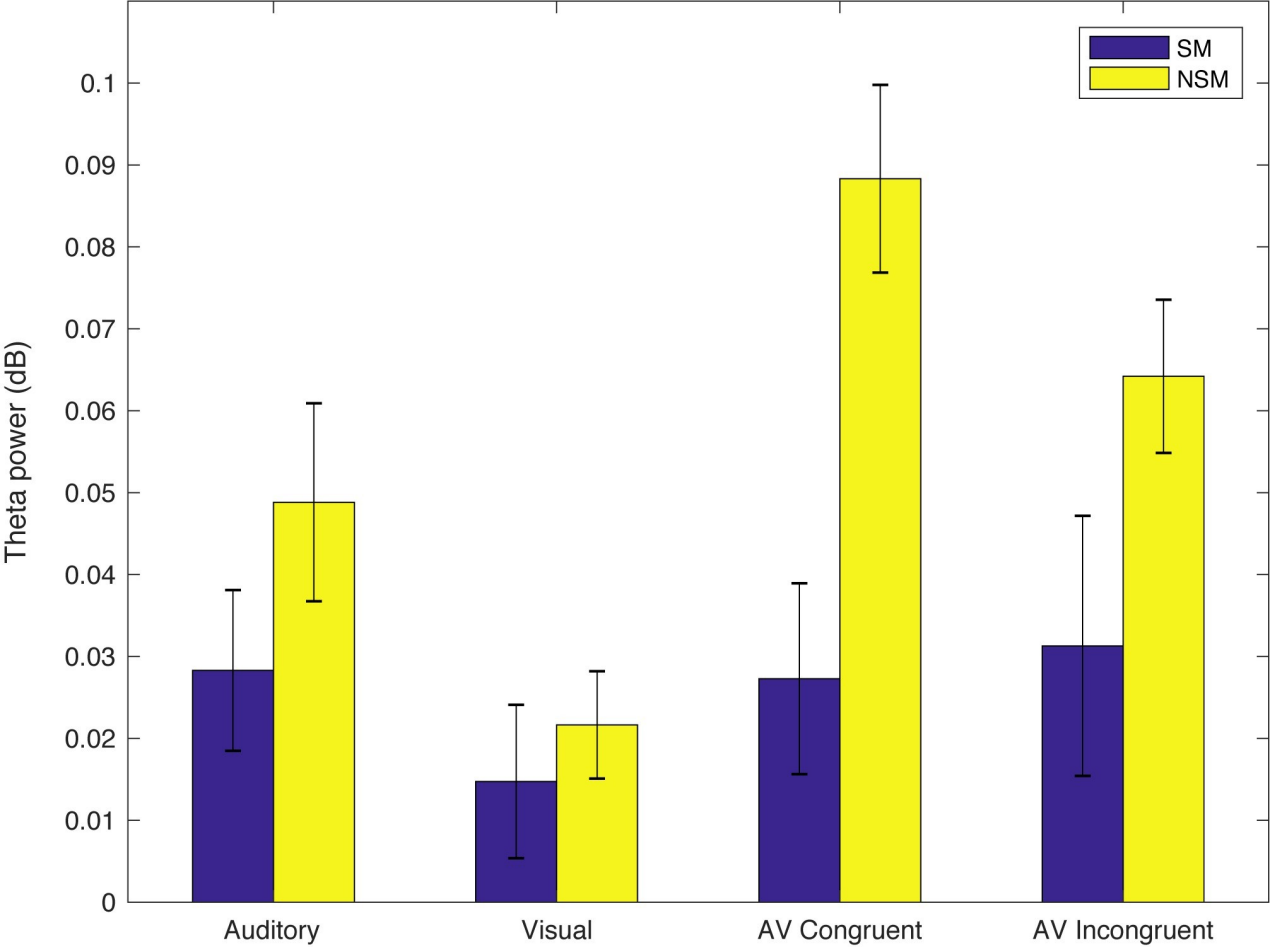
Mean power over the SM < NSM cluster found for the pooled audiovisual conditions. Whiskers represent the standard error of the mean over participants.

We wanted to further investigate whether the effect found for the audiovisual congruent condition is a general audiovisual effect or rather a specific effect of congruence, by doing a two-way ANOVA in addition to the pairwise direct comparisons done in the permutation tests. In order to do that, we first pooled the congruent and incongruent trials, subtracted out the evoked potential and ran a cluster-based permutation test comparing SM to NSM theta power in the same fashion as for the single conditions. The (time, frequency, sensor) distribution of the cluster found for the pooled AV conditions (p = 0.0342) was subsequently used as a mask to compute the mean power over the cluster for each subject and condition. The mean theta power for each group and condition is shown in Fig 4.

A 2 (Group; Speech mode, Non-speech mode) x 2 (Condition; AV Congruent, AV Incongruent) repeated-measures ANOVA that was run on the means of the audiovisual conditions revealed a main effect of Group (F(1,26) = 11.90, *p* = 0.0019), but no effect of Condition (F(1,26) = 0.86, p = 0.37) or Group X Condition interaction (F(1,26) = 1.24, *p* = 0.21). Thus, there were no statistical differences between the congruent and incongruent audiovisual conditions within the specified cluster.

### MMN dataset: No McGurk-MMN related differences in induced theta power

If the theta-band effect is the result of a general mismatch mechanism, it should be observable in the McGurk-MMN data set: theta power should be higher for deviant trials than for standard trials. Also, if the effect is the result of a specific audiovisual mismatch detection mechanism, theta power should be higher for deviant trials as they are also audio-visually incongruent whereas the standard trials are not. Hence, the comparison of standard vs deviant trials in SM tests for both types of mismatch effects. Both audiovisual incongruence and the perceptual difference between standard and deviant trials should only occur in the Speech mode group but not the Non-speech mode group [35], thus the deviant > standard difference would be expected for SM but not NSM.

**Fig 5:**
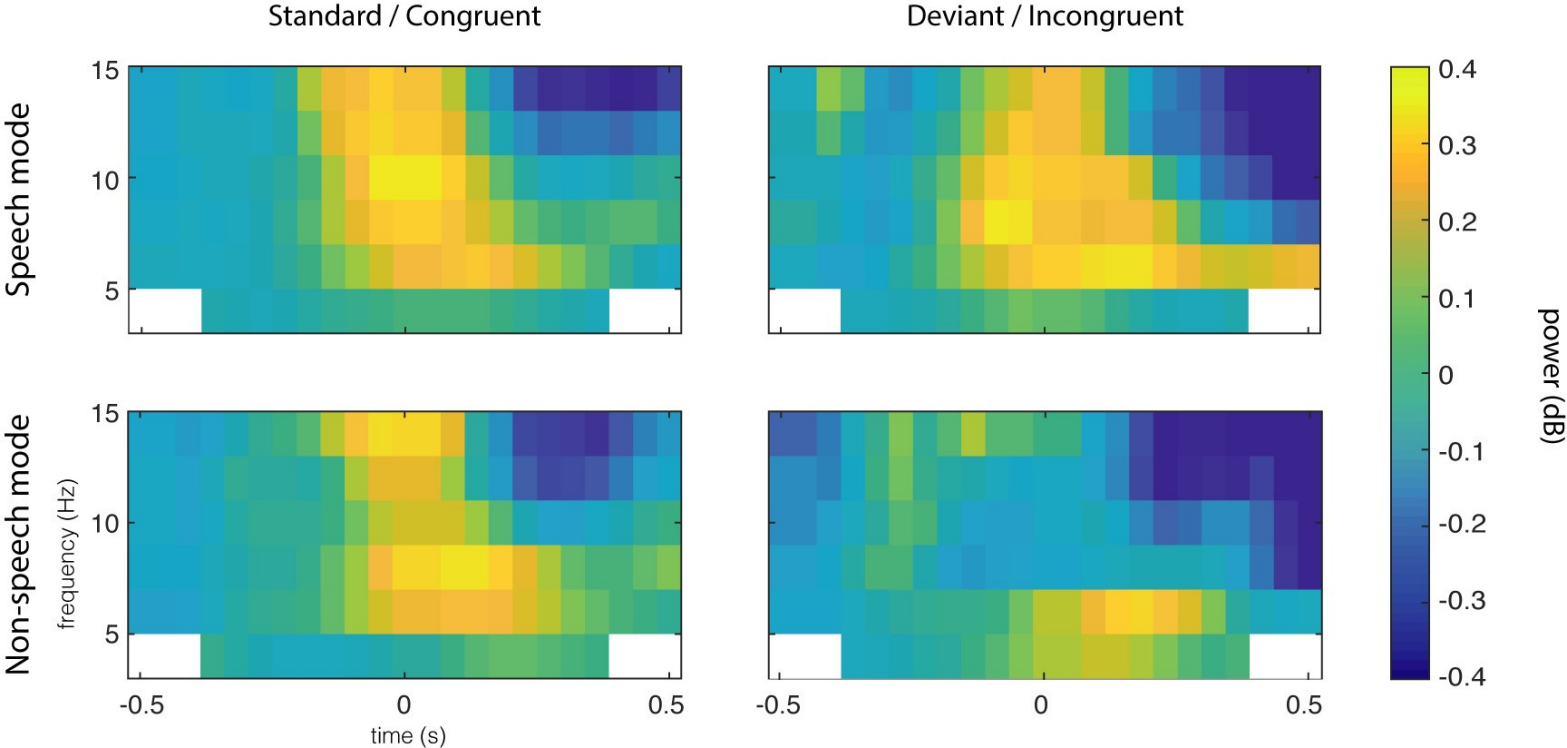
Grand average power at a central sensor for the MMN dataset, by group and condition. For the Speech mode group, there are no clear differences between the conditions, contrary to the mismatch hypothesis. For the Non-speech mode group, there seems to be a deviant < standard difference in the alpha and upper theta band, which cannot be explained by any of our hypotheses.

**Fig 6:**
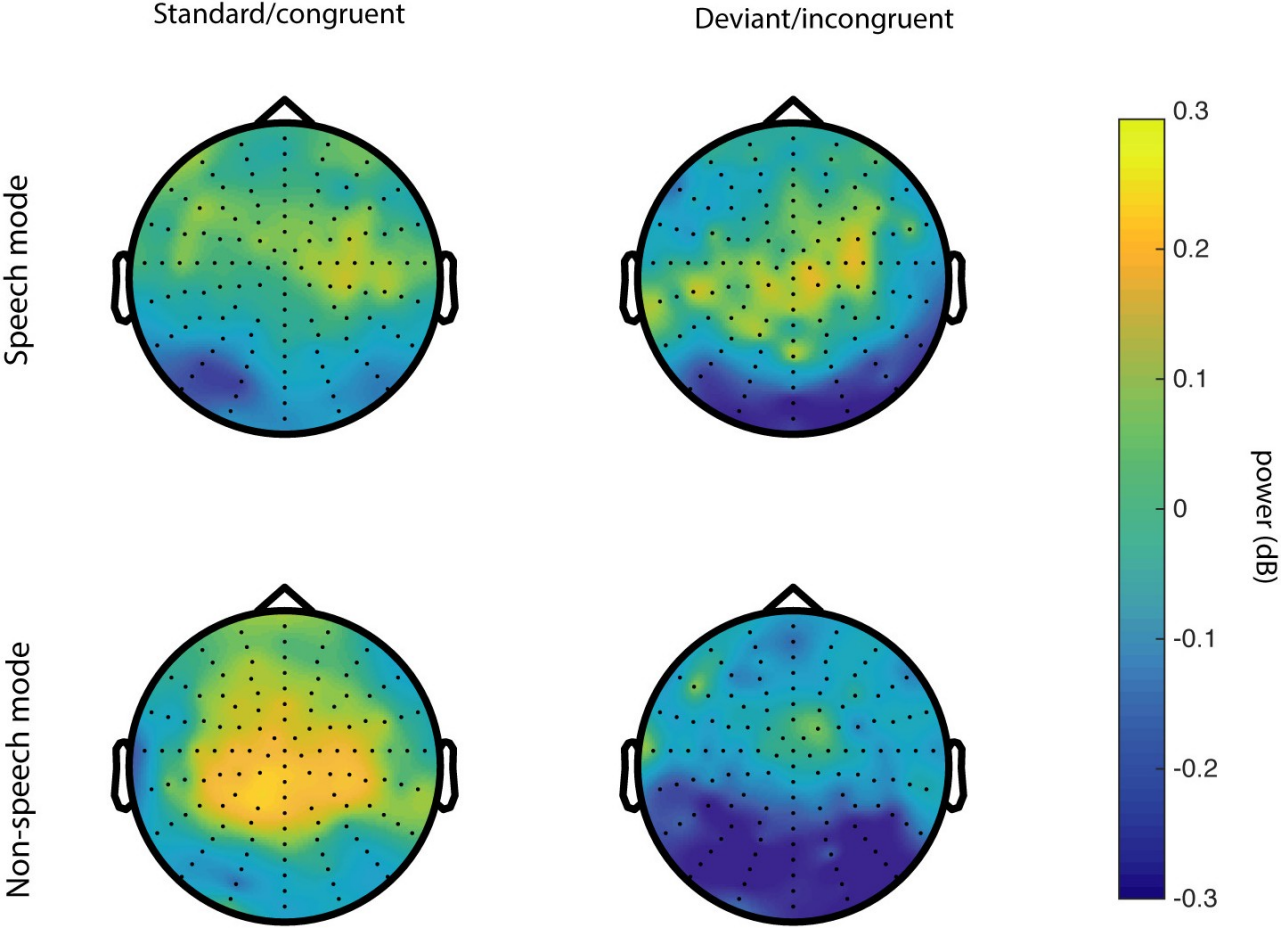
Topographic distribution of grand average 4–8 Hz power for speech mode (left) and non-speech mode (right) at 200–500 ms.

A one-tailed cluster-based permutation test of the deviant > standard hypothesis was run on 4–8 Hz from the time point of visual difference in the stimuli (140 ms, corresponding to onset of /m/ and /n/, respectively) up to 500 ms. No significant difference was found in this direction, either for SM or NSM. Figs 5 and 6 however suggest a peculiar difference in the opposite direction (deviant < standard) for NSM; a difference that would come out as significant in a two-tailed test but does not have any obvious explanation or connection to the hypothesis.

Grand-average power is plotted by group and condition on sensor level and as topographical maps in Figs 5 and 6. As seen in Fig 6, there is a tendency toward higher power for deviant trials compared to standard trials in SM; however, this difference did not reach significance (*p* = 0.22).

The MMN dataset can also be used to test the integration hypothesis. In the standard condition we would expect a similar effect (SM < NSM in the 0–500 ms range) as in the N1/P2 dataset, but did not find any significant effects when testing this hypothesis, contrary to the findings for the N1/P2 dataset. In the deviant condition, there was no hypothesis for the SM vs. NSM contrast, since this contrast includes potential effects both of integration and mismatch. Essentially, if audiovisual integration is the only driver of the theta-band effect, we would expect SM < NSM in the deviant trials, but if it is instead driven by only mismatch processing, we would expect SM > NSM because the deviant trials elicit a mismatch signal in SM but not in NSM. Furthermore, it cannot be excluded that both effects are present, which could lead to a cancelling out since they are in opposite directions. Thus, we used a two-tailed test for the deviant trials, and found no significant differences between SM and NSM.

## Discussion

We have used EEG recordings from two SWS experiments taking advantage of the differential perceptual processing of SWS for informed compared to naïve perceivers to investigate two competing hypotheses concerning the cause of the suppression of induced theta-band oscillations first reported by Keil et al [30]. Although the experimental paradigms of the SWS experiments differ from that of Keil and colleagues, notably in the task (passive for SWS, syllable identification in Keil’s McGurk paradigm), our results point to some interesting patterns.

In the N1/P2 dataset, we found significantly lower theta power for SM compared to NSM subjects in response to congruent and pooled audiovisual stimuli. The effect is localized at frontal and fronto-central sensors and the time frame of the effect is from 0–300 ms after stimulus onset, which largely confirms the result of Keil and colleagues [30] with a slightly different time window. This effect cannot be due to mismatch processing as the stimuli were congruent and hence, the result lends support to the integration hypothesis. The time shift of the effect compared to that of Keil's study could potentially be explained by differences in the stimuli used (/aba/, /aga/ in Keil's study vs. /tabi/, /tagi/ in the current study).

The cluster-based analyses revealed no effect of perceptual mode (SM vs. NSM) for the incongruent AV condition in isolation, but we did find a significant effect when pooling congruent and incongruent trials, and the ANOVA on the mean power over the significant cluster indicated no statistical differences between the congruent and incongruent conditions in the (sensor, time, frequency) window of interest. As can be seen in Fig 4, the lack of effect for incongruent stimuli might be due to the SM versus NSM difference in the AVI condition was trending towards significance, but remained below threshold when assessed in isolation. Another possible explanation could be that the phonetic incongruence in the AVI condition did indeed elicit a mismatch signal increasing theta power for SM but not for NSM (where no phonetic incongruence was perceived) and thus cancelling out an effect of perceptual mode. However, since congruent and incongruent stimuli were identical for the first 372 ms and no ERP effects were found in the first 500 ms, if such a mismatch signal occurred it was likely not captured within the analysed time interval of 0–500 ms after sound onset. Hence, although found no difference between the congruent and incongruent conditions in our data, this result does not generalize to other paradigms. We believe that the congruent vs. incongruent contrast would be informative, and should be a subject of future studies.

In the MMN dataset, we did not find any of the expected effects in theta-band oscillatory power, either in relation to the McGurk-MMN (standard vs. deviant trials in SM), or between the groups. Thus, in these data we do not find support for the mismatch hypothesis, which proposed that the theta-band suppression as observed by Keil et al [30] is an effect of mismatch processing. Although the MMN component of the ERP translates to enhanced total theta-power [47], the same does not necessarily apply for the induced oscillations. It has been shown that the auditory MMN is accompanied by increased phase-locking of the theta-band activity [47–49]; thus, the audiovisual mismatch signal may also well be characterized as a synchronization of ongoing oscillations resulting in a larger proportion of evoked compared to induced oscillations, rather than an amplification of total oscillatory activity. Notably, the deviant stimulus in the McGurk-MMN paradigm additionally requires AV phonetic incongruence processing, as it is incongruent and produces an auditory illusion. The oddball paradigm used for the MMN dataset cannot isolate this AV-mismatch processing from the memory-related MMN, and thus it cannot be ruled out that these two effects somehow interact to cancel out.

The lack of between-groups effects in the Mismatch data set for both standard and deviant trials seems to contradict the results found in the analysis of the N1/P2 dataset. This may possibly be explained by repetition effects caused by the fixed ISI in this experiment. Predictable timing of sound onset has well-documented effects on the ERP components on similar latencies as the theta-band effect, for example the N1/P2 complex [50]. In the time/frequency domain, effects of predictive timing have been found on oscillations in various frequency bands [51]. This could explain the qualitative differences between the time-frequency maps in Figs 2 and 5, where a broadband activation is seen at sound onset for the fixed ISI MMN experiment in Fig 5, but absent in the variable ISI ERP experiment in Fig 2. This observation raises the more general question of whether the observed effect on theta-band power, which we argue to be a neural correlate of audiovisual integration of phonetic features, is replicable in a more naturalistic setting. In natural speech, syllables and words are delivered quasi-rhythmically and thus the onset of speech tokens is at least to some extent predictable, although more variable than the fixed ISI used in the MMN dataset. This question cannot be addressed with the data used in this study, and thus requires further studies.

In conclusion, we do not find support for the claim that the difference in induced theta-band power between McGurk fusions and non-fusions observed by Keil and colleagues [30] is caused by a mismatch signal. This negative finding can, of course, not exclude an effect of general mismatch processing on induced theta-band power, as it may be due to lack of statistical power or interactions with phase-locking effects. However, our results do suggest differences in perceptual processing–more specifically as differential audiovisual integration of the stimuli–contribute to a modulation of induced theta power. Furthermore, our experimental approach demonstrates the benefit of using perceptually ambiguous stimuli such as sine-wave speech in the study of induced cortical oscillations.

## Supporting information

S1 Fig Result of the cluster-based permutation test for the AV Incongruent condition. No significant differences were found.
(TIF)

S2 Fig Result of the cluster-based permutation test for the Auditory condition. No significant differences were found.
(TIF)

S3 Fig Result of the cluster-based permutation test for the Visual condition. No significant differences were found.
(TIF)
