# Supplementary figures and images for: Speech-specific audiovisual integration modulates induced theta-band oscillations
Source: PLoS One. 2019 Jul 16;14(7):e0219744. doi: 10.1371/journal.pone.0219744 (PMC6634411; doi:10.1371/journal.pone.0219744)

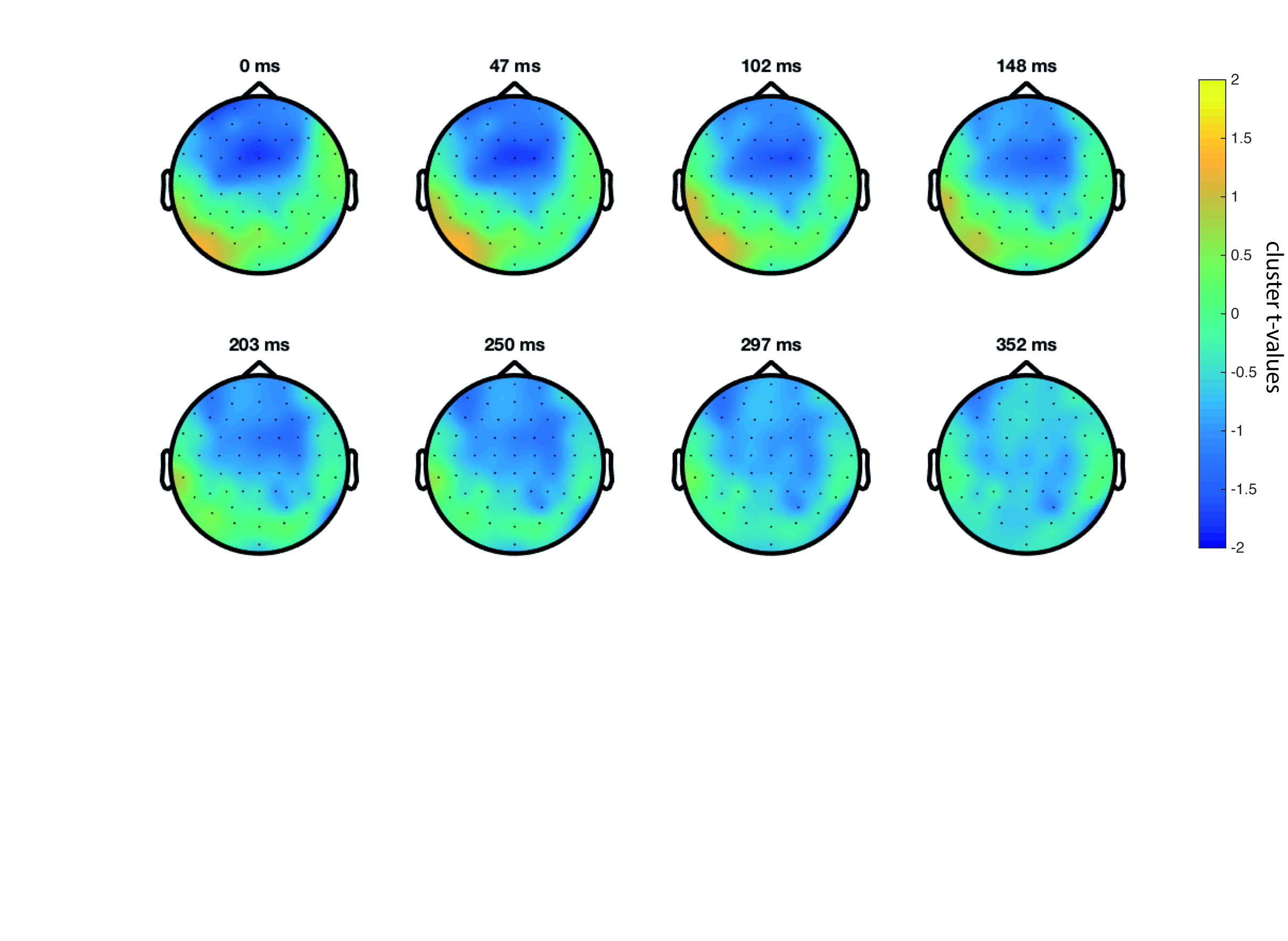

Supplement: S1 Fig — No significant differences were found. (TIF) [file pone.0219744.s001.tif]

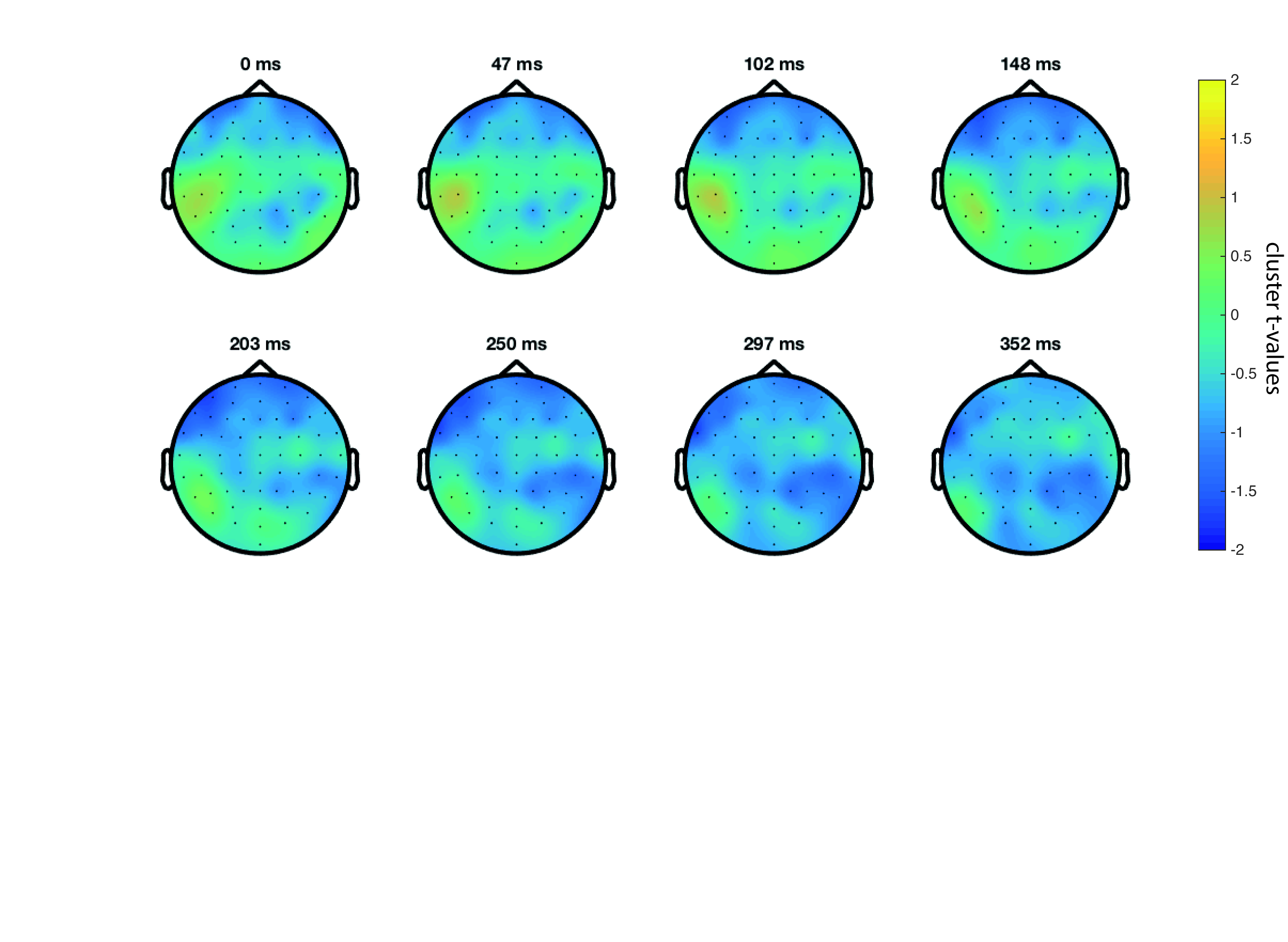

Supplement: S2 Fig — No significant differences were found. (TIF) [file pone.0219744.s002.tif]

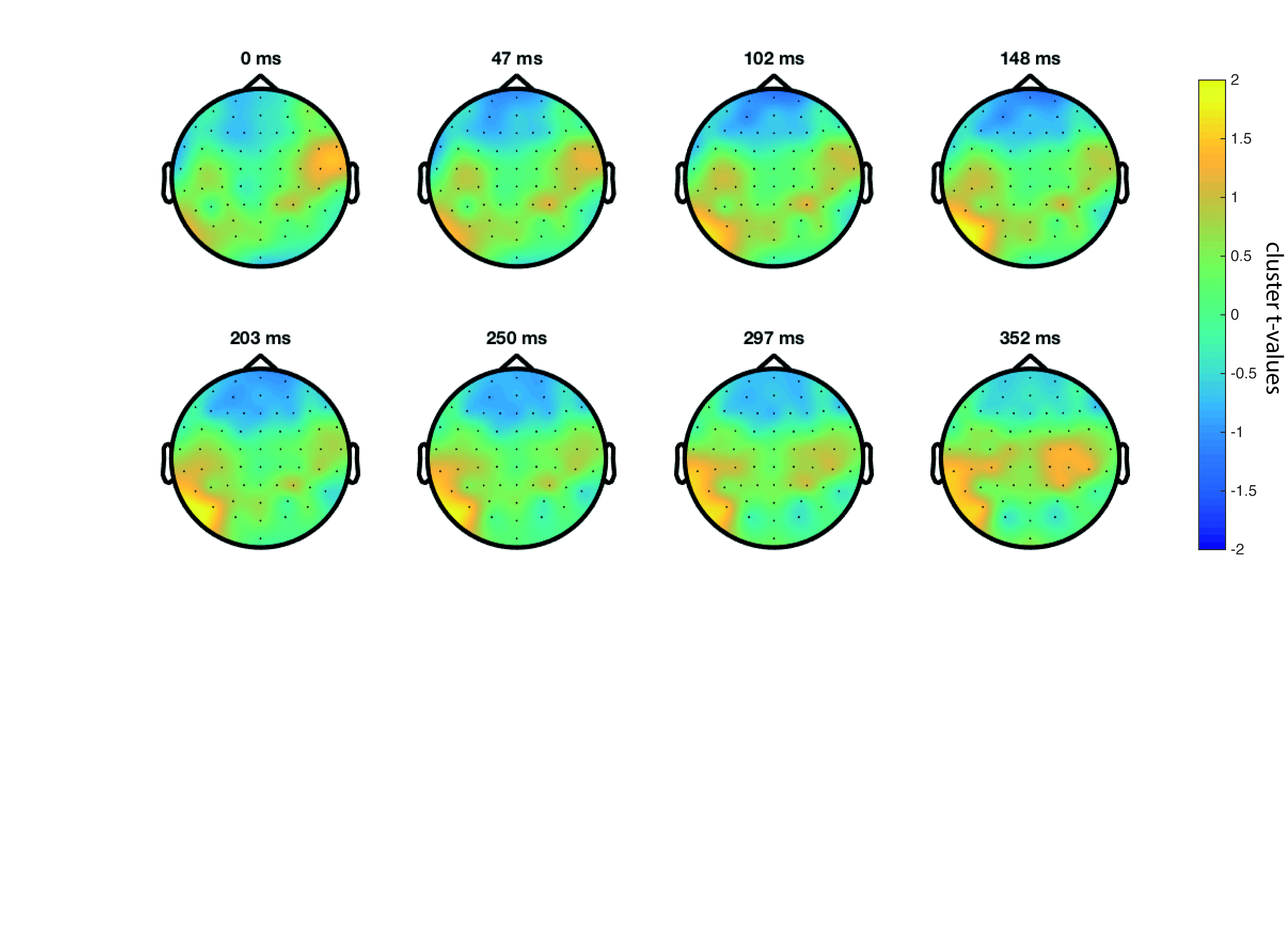

Supplement: S3 Fig — No significant differences were found. (TIF) [file pone.0219744.s003.tif]
